# The Pathological Mechanism and Potential Application of IL-38 in Autoimmune Diseases

**DOI:** 10.3389/fphar.2021.732790

**Published:** 2021-09-02

**Authors:** Miao-miao Han, Xin-rong Yuan, Xiang Shi, Xing-Yu Zhu, Yue Su, De-Kai Xiong, Xing-Min Zhang, Huan Zhou, Ji-Nian Wang

**Affiliations:** ^1^ School of Health Management, Anhui Medical University, Hefei, China; ^2^ Department of Neurology, Xiangya Hospital, Central South University, Changsha, China; ^3^ School of Pharmacy, Bengbu Medical College, Bengbu, China; ^4^ National Drug Clinical Trial Institution, The First Affiliated Hospital of Bengbu Medical College, Bengbu, China; ^5^ Public Basic College, Bengbu Medical College, Bengbu, China; ^6^ Department of Education, The First Affiliated Hospital of Anhui Medical University, Hefei, China; ^7^ Department of Epidemiology and Biostatistics, School of Public Health, Anhui Medical University, Hefei, China

**Keywords:** IL-38, autoimmune diseases, inflammation, genome editing, ra

## Abstract

Interleukin-38 (IL-38), a new cytokine of interleukin-1 family (IL-1F), is expressed in the human heart, kidney, skin, etc. Recently, new evidence indicated that IL-38 is involved in the process of different autoimmune diseases. Autoimmune diseases are a cluster of diseases accompanied with tissue damage caused by autoimmune reactions, including rheumatoid arthritis (RA), psoriasis, etc. This review summarized the links between IL-38 and autoimmune diseases, as well as the latest knowledge about the function and regulatory mechanism of IL-38 in autoimmune diseases. Especially, this review focused on the differentiation of immune cells and explore future prospects, such as the application of IL-38 in new technologies. Understanding the function of IL-38 is helpful to shed light on the progress of autoimmune diseases.

## Introduction

Autoimmune diseases are resulted from a chronic immune response to the host’s own cells, tissues, and organs and then lead to dysfunction, tissue destruction, and pathological changes (Eggenhuizen et al., 2020). According to the location of the lesion, autoimmune diseases are classified into systemic and organ-specific autoimmune diseases (Gao et al., 2021). In 2020, autoimmune diseases affected about 5–8% of the global population (Fugger et al., 2020). Genetic (Jonkers and Wijmenga, 2017), epigenetic (Jeffries, 2018), environmental (Ilchmann-Diounou and Menard, 2020), hormonal factors (Xiao et al., 2021) are considered to affect the process of autoimmune diseases. Immune cells, like T lymphocytes (Khan and Ghazanfar, 2018), B cells (Rubin et al., 2019), natural killer (NK) cells (Gianchecchi et al., 2018), and inflammatory cytokines, for instance, interleukin-1 (IL-1) (Migliorini et al., 2020), IL-15 (Patidar et al., 2016), IL-35 (Su et al., 2018), are involved in autoimmune diseases. The changes of immune cells and inflammatory cytokines stimulated by environmental factors and other risk factors may affect the occurrence of autoimmune diseases (Sumida et al., 2018; Yasuda et al., 2019). Currently, the pathogenesis of most autoimmune diseases is unclear (Wu et al., 2020), but some new immune cytokines are attracting more and more attention.

IL-38 is a novel cytokine. Similar to IL-1 receptor antagonist (IL-1Ra) and IL-36Ra, IL-38 is a typical receptor antagonist of IL-36 antagonist (van de Veerdonk et al., 2012). It is usually expressed in the skin (Han et al., 2019), spleen (van de Veerdonk et al., 2018), synovial (Boutet et al., 2019), etc. in healthy humans. Also, IL-38 is expressed in the B cells proliferating in skin basal epithelium and tonsils (Lin et al., 2001). Furthermore, IL-38 might exert anti-inflammatory effects by reducing the production of pro-inflammatory cytokines secreted by synovial fibroblasts and macrophages (Boutet et al., 2017). In addition, it could inhibit the production of T-cell cytokines IL-17 and IL-22 (Yuan et al., 2015). Notably, the expression level of IL-38 is abnormal in rheumatoid arthritis (RA) patients, and IL-38 can restrain inflammatory responses in collagen-induced arthritis (CIA) mice via Sirtuin1/Hypoxia-inducible factor-1α (SIRT1/HIF-1α) signaling pathway (Xu et al., 2018; Pei et al., 2020). Overall, IL-38 might participate in regulating autoimmune diseases.

More evidences showed that IL-38 might play roles in the balance of immune cells with regulation of cytokines as a mechanism to participate in autoimmune diseases, suggesting that IL-38 may impact autoimmune diseases (Mora et al., 2016; Xie et al., 2019). This review discussed IL-38 and autoimmune diseases, as well as the function and regulatory mechanism of IL-38 in autoimmune diseases. PubMed and ScienceDirect electronic databases were searched systematically without restricting the languages and year (up to 2021). Search terms included “IL-38” combined with “autoimmune diseases” or “inflammation” or “systemic lupus erythematosus (SLE)” or “RA” or “psoriasis” or “inflammatory bowel disease (IBD)” or “autoimmune thyroid disease (AITD)” or “multiple sclerosis (MS)” or “primary Sjogren’s syndrome (pSS)” or “Behcet’s disease (BD)” or “genome editing.” Particularly, this reviewpaid attention on that IL-38 may regulate autoimmune diseases by regulating immune responses, which will help to understand IL-38 and provide an abundant basis for clinical treatment and drug research.

## Overview of IL-38

**FIGURE 1:**
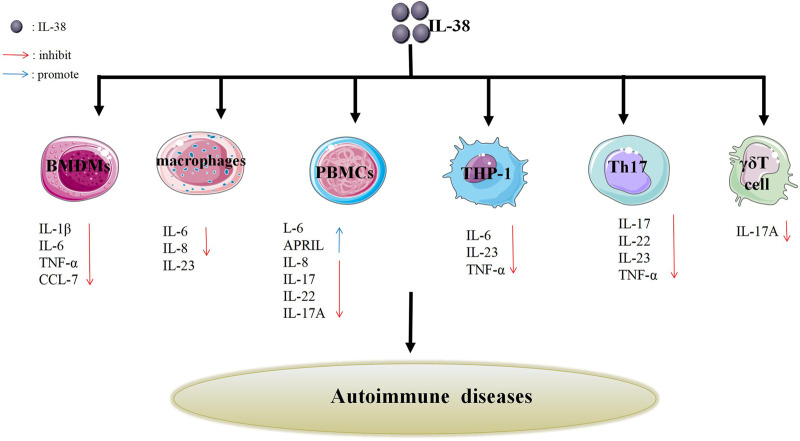
Effects of IL-38 on different cells. IL-38 affects the secretion of different cells such as Th17 cells, γδT cells, PBMCs, BMDMs, macrophages, THP-1 cells, etc., thereby inhibiting the production of related cytokines (IL-17, IL-22, IL-23, etc.) or promoting the production of related cytokines (IL-6 and APRIL). The inhibition or promotion effect may impact autoimmune diseases.

IL-38, a newly discovered cytokine, is located on chromosome 2q13-14.1 (Gianchecchi et al., 2018). It was identified by researchers in 2001 using a high-throughput cDNA sequence and renamed in 2010 (Dinarello et al., 2010; Xie et al., 2019). IL-38 has a molecular weight of 16.9 kd, lacks a signal peptide, and consists of 5 exons. The most common amino acids are glutamic acid, alanine, and leucine; followed by glycine, proline, and serine (Yuan et al., 2015). Besides, IL-38 is part of IL-1family (IL-1F). And IL-1F is composed of anti-inflammatory and pro-inflammatory cytokines, for instance, IL-37, IL-1β (Dinarello, 2019). On the one side, IL-37 could transform the cytokine expression from pro-inflammatory to anti-inflammatory via regulating macrophage polarization and lipid metabolism (Yang et al., 2019). On the other side, IL-1β could directly activate the gammadeltaT17 (γδT17) cells in mice and stimulate keratinocytes (KCs) to secrete chemokines, resulting in inflammatory responses and then leading to skin inflammation (Cai et al., 2019). In view of the above-mentioned facts, IL-38 might have anti-inflammatory or pro-inflammatory effects like its family members (Figure 1).

Broadly speaking, IL-38 is secreted by various immune cells, for instance, B cells (Xie et al., 2019). Notably, as a B cell product, the relative deficiency of IL-38 is related to increased systemic inflammation in metabolic diseases, cardiovascular, and aging (de Graaf et al., 2021). Generally, IL-38 is expressed in human heart, thymus etc. (Bensen et al., 2001), but not in T cells in the tonsil (Kumar et al., 2000). Also, it was abnormally expressed in colonic tissue of colorectal cancer (CRC) (Chen et al., 2020). The CRC patients with high level of IL-38 versus the low level groups had a longer survival time. In addition, in the antibody-induced arthritis mouse model, IL-38-deficient mice performed a high degree of joint inflammation than wild-type mice (Takenaka et al., 2015). This data showed that IL-38 might be related to different diseases.

There are many immune cells and signaling pathways in mechanism that may regulate the diseases progression via IL-38. For instance, IL-38 can promote tumor growth in lung cancer tumor microenvironment via regulating CD8^+^ tumor-infiltrating lymphocytes (Kinoshita et al., 2021). It suggested that IL-38 may participate in lung cancer by regulating immune cells. Additionally, IL-38 binds to the receptors via nuclear factor kappa-B (NF-κB), activating protein-1 (AP-1), and c-Jun N-terminal kinase (JNK) signaling pathways to regulate the inflammatory cytokines generation (Xu and Huang, 2018). This data indicated that IL-38 might be related to autoimmune diseases. Furthermore, IL-38 may affect the mechanism of autoimmune diseases in regulating the balance of anti-inflammatory and pro-inflammatory.

## Overview of Autoimmune Diseases

Autoimmune diseases are due to the loss of immune tolerance to self-antigens, leading to immune responses to self-organizations (Watad et al., 2017). More than 100 kinds of autoimmune diseases have been differentiated (Vangoitsenhoven and Cresci, 2020). Autoimmune diseases are classified into organ-specific [primary biliary cirrhosis (PBC)] and systemic autoimmune diseases (RA) (Jeffries, 2018). In 2020, autoimmune diseases affected about 5–8% of the global population (Fugger et al., 2020). The cause of autoimmune diseases is multifactorial. Genetics, environment and immune responses are considered to be relevant to the progression of autoimmune diseases (Li et al., 2019).

Generally speaking, chronic and intermittent inflammation and the destruction of tolerance are the main pathogenesis of autoimmune diseases, in which B and T cells may be involved (Singh et al., 2012; Gianchecchi et al., 2013; Gupta and Louis, 2013; Romo-Tena et al., 2013; Khan and Ghazanfar, 2018; Barnas et al., 2019). On the one side, continuous stimulation of dendritic cells (DCs) by autoantigens will enhance the activity of B cells and drive autoreactive B cells to produce autoantibodies and pro-inflammatory cytokines (Toubi and Vadasz, 2019). On the other side, T lymphocytes are crucial to regulate the immune system (Khan and Ghazanfar, 2018). Currently, many autoimmune diseases could be regulated by various immune cells. For instance, CD22 and CD72 inhibited the proliferation of regulatory B cells, which can regulate MS and type 1 diabetes mellitus (T1D) (Tsubata, 2019). Understanding the role of various immune cells in different autoimmune diseases can help enrich treatment options.

In terms of treatment, there are many feasible methods, such as traditional drug therapy (Jiang et al., 2020), immunotherapy (Terry and Oo, 2020). For example, the IL-6 inhibitor, tocilizumab was well applied for treatments of RA (Tanaka et al., 2018). Moreover, the IL-1F, for instance, IL-18, participated in regulating the immune response in autoimmune diseases and provided opportunities for new therapies of autoimmune diseases (Migliorini et al., 2020). Like other IL-1F cytokines, IL-38 may participate in autoimmune diseases. The interleukin cytokine therapy is a kind of treatment method that is gradually increasing and pretty effective. It is also a bright way to use more and more interleukin cytokines as the research direction.

## The Function Role of IL-38 in Autoimmune Diseases

**FIGURE 2:**
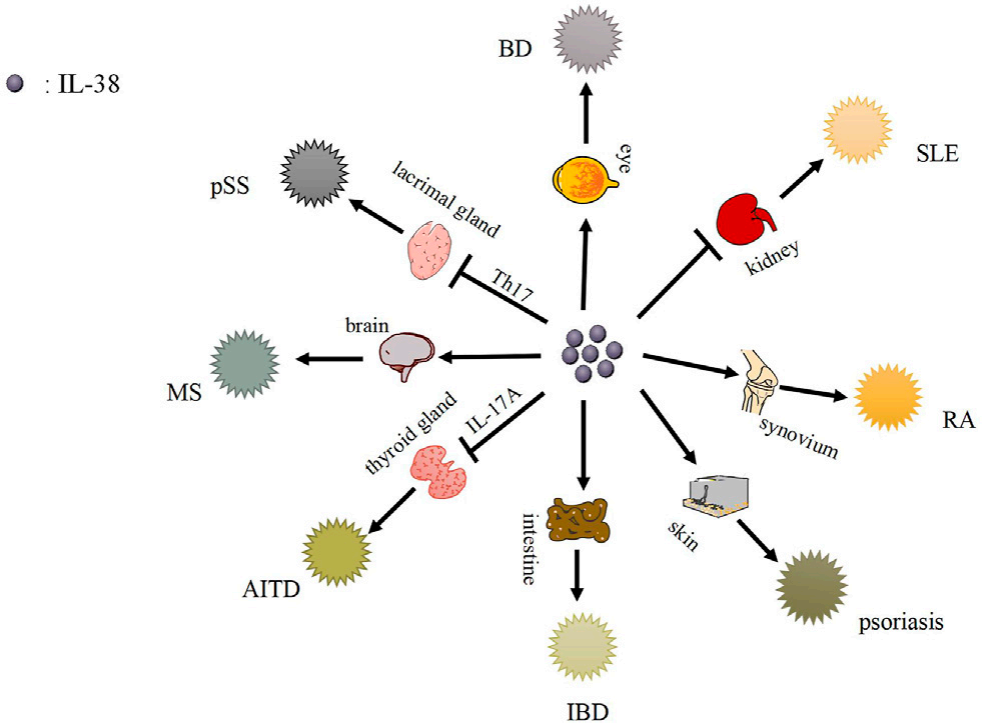
The function role of IL-38 and its related autoimmune diseases. IL-38 is expressed in kidney, synovium, skin, intestinal tissues, etc., and causes related autoimmune diseases, such as SLE, RA, psoriasis, etc.

IL-38 plays a role in immune cells, for instance, B cells and T cells, while autoimmune diseases are related to immune cells (Singh et al., 2012; van de Veerdonk et al., 2012; Romo-Tena et al., 2013; de Graaf et al., 2021). And IL-38 might impact the process of autoimmune diseases mediated via immune cells (Garraud et al., 2018). Besides, IL-38 is expressed in different autoimmune diseases accompanied by different levels, which may further affect autoimmune diseases (Tables 1–3) (Figure 2).

**TABLE 1:** The expression and function role of IL-38 in autoimmune diseases.

Autoimmune diseases	Expression level of IL-38 in serum/plasma	Function role of IL-38	References
SLE	Human (+)	IL-38 increased expression levels of IL-6 and APRIL. IL-38 decreased lupus-like clinical symptoms histopathological features of skin and nephritis.	26314375 29385862 33051219 27769564
RA	Human (+)	IL-38 decreased expression levels of IL-6, IL-23, and TNF-α. IL-38 decreased expression levels of Th17 cytokines and TNF-α.	30268016 26701127 28288964 32347300
AITD	Human (+)	IL-38 inhibited the expression of IL-17A. IL-38 inhibited inflammation.	33383445
MS	Human (+)	IL-38 inhibited IL-17-driven inflammation.	33080590 10471494 33504620
pSS	Human (−)	IL-38 inhibited the expression of IL-27 and IL-23. IL-38 decreased the frequency of Th17 cells and IL-17 protein.	32950755
BD	Human (−)	IL-38 was significantly correlated with eye involvement.	31957702

**TABLE 2:** The expression level of IL-38 in tissues of autoimmune diseases.

Autoimmune diseases	The expression level of IL-38 in tissues				Subjects	References
RA	Synovium (+)				Human (+) mice (+)	26701127 31504934
Psoriasis		Skin (−)			Human (−) mice (−)	31387327 30995480 26701127
IBD			Mucosa (+) serosa (+) submucosa (+) muscular layer (+) intestine (+)		Human (+) mice (+)	30643810 31927461
AITD				Circulating and orbital connective (−)	Human (−)	33693700

**TABLE 3:** The expression level of IL-38 in cells of autoimmune diseases.

Autoimmune diseases	The expression level of IL-38 in cells			Subjects	References
SLE	PBMCs (+)			Human (+)	33079487
MS		Macrophages (+)		Mice (+)	33504620
pSS			Acinar epithelial cells (+) infiltrating mononuclear cells (+)	Human (+)	25902739

### SLE

SLE, an incurable autoimmune disease, is due to inappropriate immune responses to nucleic acids containing cellular particles from the innate and adaptive immune system (Qiu et al., 2021). The deposition of autoantibodies in kidney, skin, and lung is a characteristic of SLE (Bialas et al., 2017). The clinical manifestations of SLE are quite diverse, but common ones mainly include rash, arthritis, and constitutional symptoms. On the other side, SLE patients perform severe organ-threatening complications, like lupus nephritis (Dorner and Furie, 2019). In 2021, the incidence rate is 0.3–31.5 per 100,000 people per year and the adjusted prevalence worldwide is approaching 50–100 per 100,000 people (Fanouriakis et al., 2021). In addition, there are evidences to support the close relationship between IL-1F cytokines and SLE. For instance, IL-33 neutralization can suppress lupus disease in lupus-prone mice, indicating that IL-33 blockade has protective effects on SLE (Li et al., 2014). As a novel cytokine, IL-38 might participate in the process of SLE.

A research demonstrated that the serum level of IL-38 was expressed in SLE patients and the concentration of IL-38 was related to the risk of renal and central nervous system (CNS) complications (Rudloff et al., 2015). However, Takeuchi et al. (2018) indicated that only one of the 19 juvenile-onset patients had elevated IL-38 level and the level was decreased after treatment. Nevertheless, specific datas were not shown. Furthermore, Xu et al. (2020a) reported that compared with volunteers, the mRNA level of IL-38 in peripheral blood mononuclear cells (PBMCs) of SLE patients was increased. *In vitro*, silencing endogenous IL-38 in PBMCs caused an apparent augment in the lupus-associated mediators IL-6 and APRIL (a proliferation-inducing ligand) (Fanouriakis et al., 2021). Additionally, serum level of IL-6 significantly was increased in active patients (Thanadetsuntorn et al., 2018). Also, elevated urine IL-6 level in SLE patients was related to disease activity and the presence of active urine sediment. The level of IL-6 in SLE active urinary sediment patients was higher than normal or inactive urinary sediment patients (Peterson et al., 1996). Of note, Stanescu et al. (2018) showed that saliva may be utilized to monitor IL-6 level and inflammatory status in SLE patients. In conclusion, IL-6 is of great significance in SLE. On the other hand, APRIL level was significantly correlated with proteinuria and renal histological activity index (Samy et al., 2017). Patients with elevated serum APRIL level had severe proliferative glomerulonephritis, for instance, fibrinoid necrosis (Treamtrakanpon et al., 2012). This data suggested that APRIL may be useful for further study of SLE.

In Murphy Roths Large (MRL)/lpr model mice, the expression level of IL-38 was decreased in spleen and thymus than control groups (Chu et al., 2017). After IL-38 treatment, lupus-like clinical symptoms histopathological features of skin and nephritis in mice were relieved. It was observed that the inflammatory infiltration in the stained skin sections of mice treated with IL-38 was decreased. In addition, the glomerulonephritis score of IL-38 treatment group was significantly decreased, mainly manifested as the improvement of glomerular mesangial thickening and proliferation (Chu et al., 2017). In brief, IL-38 can alleviate the symptoms in SLE mice and may have a protective effect on SLE.

### RA

RA is a chronic immune-mediated disease, in which various immune cells and signaling networks malfunction to trigger a maladaptive tissue repair process leading to organ damages and various degrees of disability (Pandolfi et al., 2020; Weyand and Goronzy, 2021). Joint dysfunction, cartilage injury, and debilitating pain are typical symptoms of RA (Magyari et al., 2014). In 2021, Hansildaar et al. (2021) have stated that the prevalence of RA is up to 1%. Although the development and progression of RA is still not completely clear, different interleukin cytokines, for instance, IL-6 (Pandolfi et al., 2020) and IL-37 (Wu et al., 2021) are involved in RA. As an interleukin cytokine, IL-38 may get involved in RA.

Recently, Xu et al. indicated that serum level of IL-38 in RA patients was abnormal (Xu et al., 2018). Moreover, compared to osteoarthritis (OA) and psoriasis arthritis (PsA), the expression level of IL-38 was higher in plasma, synovial fluid, and synovium of RA patients (Boutet et al., 2016; Boutet et al., 2020). *In vitro*, after IL-38 overexpression, the levels of IL-6, IL-23, and tumor necrosis factor-α (TNF-α) in human acute monocytic leukemia cell line (THP-1) were decreased (Boutet et al., 2017). In addition, in CIA mice, the mRNA expression level of IL-38 in joints significantly was increased (Boutet et al., 2016). Moreover, after injection of adeno-associated virus IL-38 (AAV IL-38) into the joints of CIA and serum transfer induced arthritis (STIA) mice, the clinical inflammatory score was decreased significantly, accompanied by decreased macrophage infiltration and decreased expression levels of IL-17, IL-22, IL-23, and TNF-α (Boutet et al., 2017). Overall, IL-38 may influence the pathogenesis of RA, but the specific mechanism needs further exploration.

### Psoriasis

Psoriasis, an inflammatory disorder mediated by chronic immune, is featured by skin changes and systemic manifestations (Greb et al., 2016). Plaque psoriasis, inverse psoriasis, guttate psoriasis, erythrodermic psoriasis, and pustular psoriasis are five types of psoriasis (Ghoreschi et al., 2021). In 2021, the prevalence of psoriasis is known in only 19% of the countries in the world and the distribution is uneven in different geographical regions. The overall prevalence rate ranges from 0.1% in Asia to 1.5% in Europe. And the incidence of psoriasis is decreased but the prevalence rate is increased over time (Griffiths et al., 2021). In addition, psoriasis susceptibility is mainly attributed to environmental effects and genetic variation (Nussbaum et al., 2021). Although the pathogenesis of psoriasis is not completely clear, interleukin cytokines may influence in the process of psoriasis, such as IL-17 cytokines (Prinz et al., 2020). Further research on the relationship between interleukin cytokines and psoriasis, such as IL-38 and psoriasis, may help to ameliorate the process of psoriasis.

Recently, it is reported that the expression level of IL-38 was increased in normal skin but significantly decreased in peripheral blood and skin of psoriasis patients (Xie et al., 2019). Moreover, IL-38 was secreted by PBMCs and correlated with psoriasis severity (Kim et al., 2016). By restoring the physiological process of KCs proliferation and differentiation and reducing the expression of vascular endothelial growth factor A (VEGF-A), IL-38 remarkably alleviated the severity of psoriasis like phenotype induced by imiquimod (IMQ) (Mercurio et al., 2018). *In vitro*, IL-38 could inhibit Candida-induced IL-17/IL-22 and IL-36γ-induced IL-8 and have a protective role in PBMCs from healthy donors (van de Veerdonk et al., 2012). It can be clearly observed that in the presence of IL-38, the production of IL-17A and IL-22 induced by Candida, as well as IL-8 induced by IL-36γ was decreased, respectively. Furthermore, IL-22 may exert an anti-apoptotic effect on KCs to balance cell proliferation and apoptosis in psoriasis epidermis (Wang et al., 2020). In addition, IL-38 was abnormally expressed in mouse skin (Han et al., 2019). In psoriasis mouse model induced by IMQ, Boutet et al. (2016) found that the expression levels of IL-36α, IL-36γ, and IL-36Ra were increased at the peak of psoriasis while IL-38 level was decreased. Notably, mRNA expression level of IL-38 was negatively related to psoriasis area and severity index (PASI) and IL-17A, but positively correlated with cytokeratin 10 (CK10) expression. To sum up, the expression level of IL-38 in psoriasis is decreased, which may become a biomarker of psoriasis biological diagnosis.

### IBD

IBD, characterized by chronic immune-mediated intestinal inflammation, includes ulcerative colitis (UC) and Crohn’s disease (CD) (Graham and Xavier, 2020; Liu et al., 2021). UC is confined to the colon and can lead to ulcers, severe bleeding, toxic megacolon, and fulminant colitis. Conversely, CD could influence any part of the digestive tract and lead to complications such as fibrous stenosis, fistulas, and abscesses (Chang, 2020). Currently, the number of IBD cases in the world was increased (Jakubczyk et al., 2020). In 2020, there were 3 million patients in Europe, 3 million patients in America, and more than 80,000 patients in Australia (Jakubczyk et al., 2020). Generally, genetic, and environmental factors are relevant to IBD (Sasson et al., 2021). As for the treatment of IBD, IL-37 provides a new therapeutic target for IBD (Jia et al., 2020). As a cytokine of IL-1F, IL-38 may participate in the treatment of IBD.

Fonseca-Camarillo et al. (2018) pointed that IL-38 was expressed in IBD patients, especially in the muscular layer, mucosa, submucosa, and serosa. Moreover, the expression level of IL-38 was increased both in active CD patients and inactive UC patients. *In vitro*, the recombinant IL-38 (rIL-38) significantly reduced the expression of pro-inflammatory cytokines in lipopolysaccharide (LPS)-stimulated RAW264.7 cells and bone marrow-derived macrophages (BMDMs), for instance, IL-1β, IL-6, TNF-α and triggered an anti-inflammatory effect (Xie et al., 2020). In addition, in the dextran sulphate sodium (DSS)-induced colitis mice, Xie et al. (2020) found that IL-38 was derived from B cells in the intestine and the expression level of IL-38 was remarkably higher. After rIL-38 treatment, the symptoms of DSS-induced colitis were remarkably decreased. Compared with the phosphate-buffered saline (PBS) treatment group, DSS-induced colitis mice given rIL-38 significantly improved colonic inflammation and structural damage, including weight loss, colon shortening, and disease activity index reduction, accompanied by the reduced expression levels of TNF-α and IL-1β (Xie et al., 2020). Overall, IL-38 might become a promising therapeutic choice for IBD.

### AITD

AITD, an organ-specific autoimmune disorder, is caused by an immune attack on the thyroid due to a disorder of immune system (Antonelli et al., 2015). The main manifestations of AITD are Graves’s disease (GD) and Hashimoto’s thyroiditis (HT). Furthermore, hypothyroidism and thyrotoxicosis represent respectively the clinical hallmarks of HT and GD (Ferrari et al., 2020). AITD is attributable to the interaction of genetics and environment (Yin et al., 2020). In 2021, the prevalence of AITD accounts for about 5–20% of the whole population (Turan et al., 2021). This makes AITD one of the most prevalent autoimmune diseases (Aversa et al., 2019). The treatment of AITD has been continuously explored. It is reported that IL-1β cytokine provides the target for the developing therapeutic treatment of AITD (Zhao et al., 2013). IL-38, which belongs to the same family as IL-1β, may be related to AITD.

A study revealed that IL-38 was expressed in thyroid-associated ophthalmopathy (TAO) patients, and the expression level was increased (Pan et al., 2021). However, the expression level of IL-38 was reduced in the circulating and orbital connective tissues of TAO patients than the control group. Besides, *in vitro*, the increased IL-38 in TAO patients can inhibit the expression of IL-17A and IL-23R in PBMCs and inhibit inflammation in orbital fibroblasts (OFs) (Pan et al., 2021). It was obviously observed that when IL-38 was at a relatively low concentration, it can inhibit the secretion of IL-17A in PBMCs induced by IL-23R. However, when IL-38 was at a concentration of 100 ng/ml, it cannot inhibit IL-17A secretion. Also, IL-38 at relatively low concentrations (including 25 and 50 ng/ml) significantly reduced the expression of IL-6 and IL-8 induced by IL-1β in OFs (Pan et al., 2021). This data indicated that IL-38 may affect TAO. As there are few studies on IL-38 and TAO, the function role of IL-38 in TAO is not fully understood and further exploration is needed.

### MS

MS, a chronic autoimmune inflammatory illness, can affect the CNS and result in severe disability (Gul et al., 2020). The typical clinical symptoms of MS are characterized by weakness, sensory loss, diplopia, decreased vision, etc (Cunniffe and Coles, 2021). Moreover, inflammation with demyelination and astrocyte proliferation and neurodegeneration are two pathological features of multiple sclerosis MS (Hauser and Cree, 2020). The prevalence of MS is from 5 to 300 per 100,000 people in the world in 2021 (McGinley et al., 2021). It is well known that the pathogenesis of MS is linked to environmental and genetic factors (Kadowaki and Quintana, 2020) (32650957). In addition, studies showed that IL-18, IL-37 and other cytokines may be involved in MS (Sedimbi et al., 2013; Wang et al., 2018). As a novel cytokine, IL-38 might be related to MS.

Recently, Zarrabi et al. (2021) evaluated serum level of IL-38 in MS patients and healthy controls by enzyme-linked immunosorbent assay (ELISA). The data indicated that the level of IL-38 in newly diagnosed MS patients was higher than previously treated patients and healthy control groups. Besides, IL-38 can limit IL-17-driven inflammation and high expression of one of its receptors, IL-1 receptor accessory protein-like 1 (IL-1RAPL1), in the CNS, indicating that IL-38 might have a disease-restricting role in MS (Carrie et al., 1999). On the other hand, experimental autoimmune encephalomyelitis (EAE) is a prototypical model for MS (Constantinescu et al., 2011). In EAE model, IL-38 was abnormally expressed via macrophages infiltrating the spinal cord. Furthermore, the clinical score and histological markers of EAE in IL-38-deficient mice were significantly decreased, which was accompanied by decreased infiltration of inflammatory cells, for instance, macrophages, and decreased expression of inflammatory markers in the spinal cord (Huard et al., 2021). This data showed that IL-38 may reduce inflammation and disease severity in EAE. To sum up, IL-38 may participate in the progress of MS.

### pSS

PSS, one of systemic autoimmune diseases, can influence the exocrine glands, mainly the lacrimal glands and salivary glands. Focal lymphocytic infiltration of glands is the main characteristic (Ramsubeik et al., 2020). Furthermore, pSS also has a variety of systemic manifestations, such as polyarthritis, autonomic nervous dysfunction, lung involvement, etc (Gupta et al., 2019). In general, genetic and environmental factors may affect the occurrence of the diseases (Verstappen et al., 2018). In 2020, the prevalence of pSS is 0.01–3% of the general population (Oyelakin et al., 2020). In addition, in terms of treatments, IL-37 has an anti-inflammatory effect on pSS, which may make IL-37 a new therapeutic target for pSS (Conti et al., 2020). It is well known that IL-38 and IL-37 are both antagonists of IL-1F (Boutet et al., 2019). Whether IL-38 is used for the treatment of pSS is worth exploring.

According to the report, IL-38 was expressed in pSS patients. And mRNA and protein levels of IL-38 were up-regulated in minor labial salivary glands, mainly among infiltrating mononuclear cells and acinar epithelial cells (Ciccia et al., 2015). Inversely, Luo et al. (2020) pointed that expression levels of IL-38 and Th17 cells (including IL-17 and IL-23) were decreased in pSS patients than non-pSS group and healthy control group. And IL-17 and IL-23 could induce expression of IL-38 and form a negative loop between IL-38 and Th17 responses. Furthermore, *in vitro*, it was observed that IL-38 could inhibit expression levels of IL-27 and IL-23 in Th17 cells and minor salivary gland mononuclear cells (MSGMs). In addition, in the sjögren’s syndrome (SS) mice model, treated with IL-38, the frequency of Th17 cells and IL-17 protein was obviously decreased. And Th17 inflammation obviously was inhibited by IL-38. This is consistent with a previous research (Han et al., 2019). However, it was observed that IL-38 level was increased after IL-17 treatment (Luo et al., 2020). This data indicated an intimate interaction between IL-38 and Th17 inflammation. To sum up, IL-38 may inhibit pSS mainly via Th17 inflammation.

### BD

BD, a chronic multi-system autoimmune disease, is characterized by a chronic, relapsing remitting course of clinical manifestations, such as skin rash, oral/genital ulcers (Akiyama et al., 2020; Gholijani et al., 2020). In 2021, Yildiz et al. (2021) finds that the total prevalence of BD worldwide is 10.3 per 100,000. Moreover, Northern Jordan has the highest prevalence rate (664 per 100,000 population), followed by Turkey (600 per 100,000 population), and Scotland has the lowest prevalence rate (0.3 per 100,000 population). Generally, genetic factors and immune abnormalities are considered to be the main causes of BD (Tong et al., 2019). As for the treatment, it is reported that IL-18 could impact the regulation of the initial inflammatory pathway in BD and the targeted treatment of IL-18 may constitute a promising new therapy for BD (Prasinou et al., 2020). This data indicated that IL-1F cytokines may be essential for treatments of BD. Exploring the relationship between other cytokines, such as IL-38, and BD may provide another option for treatments of Behcet’s disease.

In one study involving 81 healthy controls and 81 BD patients, Zarrabi et al. (2019) demonstrated that serum level of IL-38 in BD patients was decreased than healthy controls. However, serum level of IL-38 was increased in those patients with eye involvement (*p* = 0.046) and female patients with a positive pathergy test (*p* = 0.048). Female patients had higher serum level of IL-38 may be due to fewer male patients. Moreover, the evaluation of IL-38 and clinical manifestations stated clearly that IL-38 level was remarkably correlated with eye involvement. This data declared that IL-38 may participate in eye involvement in BD patients. Notably, serum level of IL-38 in healthy controls was higher than BD patients, indicating that IL-38 may have protective effects on BD (Zarrabi et al., 2019). In a word, IL-38 may play a significant role in BD. Nevertheless, the limited data in Behcet’s disease requires more animal models to probe the exact function and potential mechanism of IL-38 in BD.

## The Regulatory Role of IL-38 in Autoimmune Diseases

There are many pathogeneses of autoimmune diseases and many target proteins are related (Tsou and Sawalha, 2020). The regulation of different proteins involves different signaling pathways, so the regulation of signaling pathways has a very important relationship with autoimmune diseases (Banerjee et al., 2017). For example, the NF-κB signaling pathway is bound up with the inhibition of B cell differentiation and development and function of T cell (Cai et al., 2020; Blanchett et al., 2021). Also, B cells and T cells can affect the progression of autoimmune diseases (Khan and Ghazanfar, 2018; Meffre and O'Connor, 2019). It is well known that signaling pathways, for instance, NF-κB, AP-1, JNK (Yamazaki et al., 2017; Mitchell and Carmody, 2018; Yang et al., 2021) are related to autoimmune diseases.

**FIGURE 3:**
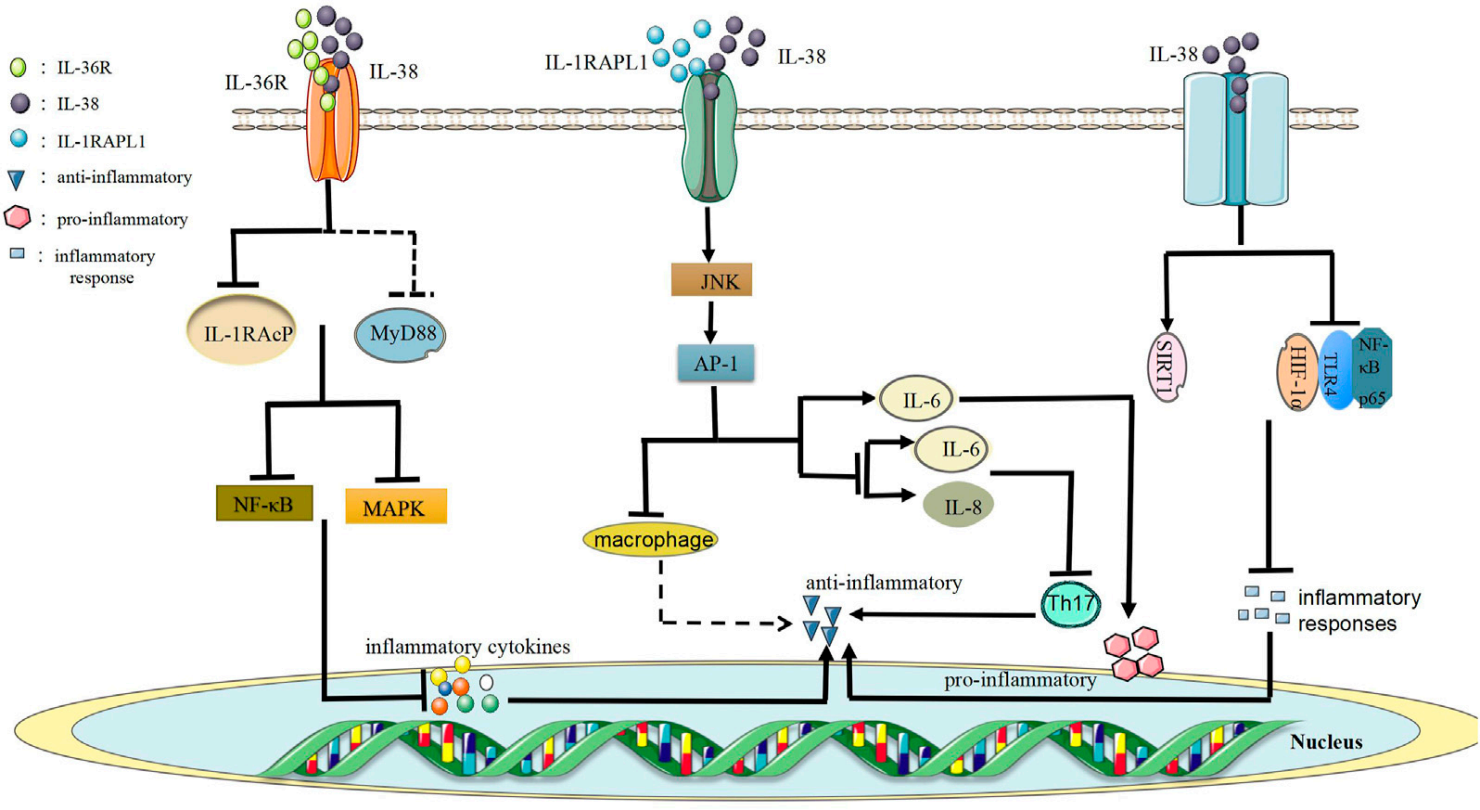
The regulatory role of IL-38 in the autoimmnue diseases.

Firstly, the low level of IL-38 protein performs as an anti-inflammatory function role via the combination of IL-36R to form the IL-38/IL-36R axis to block recruitment of the co-receptor IL-1 receptor accessory protein (IL-1RAcP) and/or it may recruit inhibitory receptors and prevent the recruitment of myeloid differentiation primary response 88 (MyD88) adaptor protein, thereby restraining NF-κB or mitogen-activated protein kinase (MAPK) signals to trigger the secretion of inflammatory cytokines (Mora et al., 2016) (Figure 3). This data indicates that IL-38 might have anti-inflammatory effects. Furthermore, the combination of IL-38 and IL-36R has inhibitory effects similar to IL-36Ra, and IL-36Ra restrains the recruitment of IL-1RAcP and blocks the signaling transduction from IL-36R (van de Veerdonk et al., 2018). Besides, IL-36Ra mutations result in the decrease of IL-36R activity that causes pustular psoriasis (Li et al., 2017). Currently, studies have supported that IL-38/IL-36R axis participates in the pathogenesis of autoimmune diseases, for example, SLE (Takeuchi et al., 2018), RA (Boutet et al., 2016), IBD (Boutet et al., 2016). To sum up, studying IL-38/IL-36R axis might provide novel choices for the treatment of autoimmune diseases.

Secondly, IL-38/IL-1RAPL1 axis is formed by the combination of IL-38 and IL-1RAPL1, which has anti-inflammatory or pro-inflammatory effects (Xie et al., 2019). For example, when the full-length IL-38 combines with IL-1RAPL1, it activates the downstream JNK/AP-1 signaling pathway and then increases the production of IL-6 to play pro-inflammatory effects. However, truncated IL-38 reduces JNK/AP-1 signals and inhibits Th17 activation to reduce inflammation macrophages activated by reducing levels of IL-6 and IL-8 (Mora et al., 2016). In addition, the production of IL-38 by apoptotic cells limits the production of cytokines in macrophages by antagonizing IL-1RAPL1-dependent JNK phosphorylation and AP-1 activation (Figure 3). Changes in macrophage cytokines can make IL-38 maintain a low Th17/Treg balance, which is beneficial to autoimmunity (Li et al., 2017). Overall, IL-38/IL-1RAPL1 axis may be vital for autoimmune diseases and the specific regulatory mechanism is worth further exploring.

Finally, SIRT1/HIF-1α signaling pathway is of great importance for RA because SIRT1 could regulate the progression of RA by interacting with HIF-1α (Pei et al., 2020). In general, SIRT protein family is consisted of seven homologous genes, namely SIRT1 to SIRT7 (Shao et al., 2016). SIRT1 is a DNA dependent protein deacetylase, which plays important roles in metabolism, immune regulation, and tumorigenesis (Hughes and Herold, 2013; Yu et al., 2018). Besides, HIF-1α, a part of the heterodimeric HIF-1 transcription factoris, is a regulatory protein and oxygen sensitive monitor in the body (Zbytek et al., 2013). Also, HIF-1α has a crucial effect on innate and adaptive immune responses and is related to inflammation and pathological activity of autoimmune diseases (Guan et al., 2017). It was shown that IL-38 restrained inflammatory responses of CIA mice via SIRT1/HIF-1α signaling pathway (Pei et al., 2020). In the CIA experiments, IL-38 up-regulated the level of SIRT1, down-regulated the levels of HIF-1α, toll-like receptor 4 (TLR4) and NF-κB p65 to inhibit inflammatory responses and alleviate joint damage in CIA mice (Pei et al., 2020) (Figure 3). This data showed that the regulatory mechanism of inhibiting inflammatory responses in CIA mice of IL-38 may be relevant to SIRT1/HIF-1α signaling pathway. Futhermore, IL-38 may participate in RA via SIRT1/HIF-1α signaling pathway. If specific regulatory role between IL-38 and SIRT1/HIF-1α signaling pathway in autoimmune diseases is clearly understood, it will provide new methods for treatments.

## Future Expectation

IL-38, a novel cytokine of IL-1F, is expressed in SLE, RA, psoriasis, IBD, and other autoimmune diseases with different expression levels of IL-38 via different signaling pathways, such as SIRT1/HIF-1α signaling pathways. Studying the function and regulatory mechanism of IL-38 in autoimmune diseases may help to provide theoretical basis and clinical methods for the treatment of autoimmune diseases. At present, some new technologies and methods have been well applied in the treatment and detection of autoimmune diseases. For example, multiplex label-free biosensor is a new proof to detect the autoantibodies in human serum diagnostics of autoimmune diseases (Orlov et al., 2020). Besides, engineered programmed death-ligand 1 (PD-L1)-expressing platelets reverse new-onset T1D (Zhang et al., 2020). Meantime, nanomaterials are used for the diagnosis and immunological imaging of T1D (Pan et al., 2020). New technologies and methods may provide a variety of treatments for autoimmune diseases.

As we all know, genome editing, an accurate and effective technology, involves DNA modifications in organisms, and includes beneficial deletions, corrections through gene replacement, and insertions (Deng et al., 2020). Besides, genome-wide association studies (GWASs) have revealed the polygenetic basis of multiple autoimmune diseases, which is essential to study the genome editing for treatments of autoimmune diseases (Pulendran and Davis, 2020). Furthermore, CRISPR/Cas9 genome editing provides a new therapeutic approach for autoimmune diseases (Mohammadzadeh et al., 2020). In fact, genome editing has been used for autoimmune diseases. For example, genetic editing to overexpress IL-37 may provide an approach to heighten the effectiveness and stability of mesenchymal stem cells (MSCs) in treating SLE (Xu et al., 2020b). IL-38, in the same family as IL-37, may be expressed in autoimmune diseases through genome editing. In the future, genome editing is likely to provide a new direction for treat autoimmune diseases by altering the level of IL-38.

In addition, as a new scientific material, nanomaterials constitute ambient ultrafine particles (UFPs) and engineered nanoparticles (ENPs) (Pollard, 2020). Nanomaterials are versatile in prevention, treatment and control of diseases (Zaheer et al., 2021). Actually, nanomaterials are beneficial to autoimmune diseases. For instance, suppressive effects of IL-27, on encephalitogenic Th17 cells induced by multiwalled carbon nanotubes, could reduce the severity of EAE (Moraes et al., 2013). Moreover, T cell-targeted nanoparticles loaded with transforming growth factor β (TGF-β) and IL-2 could induce CD4^+^ and CD8^+^ Treg cells to inhibit murine lupus (Horwitz et al., 2019). As an interleukin cytokine, IL-38 may be combined with immune cells to achieve the purpose of treating autoimmune diseases by nanomaterials. If so, it might offer a new direction and choice for the prevention of autoimmune diseases in the future.

Due to insufficient treatment options for autoimmune diseases, it is an urgent need to ameliorate the understanding of autoimmune pathogenesis so as to develop more effective methods for autoimmune diseases. Generally, current evidence supported that IL-38 may play important roles in treatments of autoimmune diseases. It may bring new directions and options for the research on autoimmune diseases.

## Conclusion

As a new cytokine, IL-38 is mainly derived from B cells and other immune cells. It is expressed in kidney, skin, etc. Moreover, IL-38 is abnormally expressed in most autoimmune diseases. The up-regulation or down-regulation expression level of IL-38 may affect different types of autoimmune diseases via different signaling pathways, for instance, SIRT1/HIF-1α signaling pathway. Furthermore, IL-38 plays anti-inflammatory and/or pro-inflammatory roles in autoimmune diseases, for instance, SLE, RA, and psoriasis. Although the current evidence supports that IL-38 participates in autoimmune diseases, the function role of IL-38 in every autoimmune disease is not fully understood. In addition, animal experimental studies on IL-38 and autoimmune diseases are still insufficient, and more animal models need to be established to probe the exact function role of IL-38 in autoimmune diseases. Finally, signaling pathways of IL-38 need a lot of exploration in autoimmune diseases to understand its regulatory role.
